# Composition of Primary Metabolites in Winter Barley Grain as Affected by NPK Fertilization of Reclaimed Land

**DOI:** 10.3390/plants15121780

**Published:** 2026-06-09

**Authors:** The Ngoc Phuong Nguyen, Minchang Kim, Jwakyung Sung, Alisdair R. Fernie

**Affiliations:** 1 Department of Crop Science, College of Agriculture, Life and Environment Sciences, Chungbuk National University, Cheongju 28644, Republic of Korea; phuongnguyen@chungbuk.ac.kr (T.N.P.N.); kimmc7263@chungbuk.ac.kr (M.K.); 2 Max-Planck-Institute für Molekulare Pflanzenphysiologie, Am Mühlenberg 1, D-14476 Golm, Germany

**Keywords:** carbon metabolism, chemical NPK fertilizer, cultivar-specific, nitrogen metabolism, winter barley

## Abstract

Optimizing nutrient management is critical for enhancing crop productivity and grain nutritional quality in reclaimed soils, where poor soil fertility and salinity often limit barley cultivation. In that context, this study evaluated the effects of NPK fertilization on barley grain metabolism in reclaimed soil, using four barley cultivars (Betaone, Heuknuri, Nurichal, and Sogang) under fertilized (F) and non-fertilized (NF) conditions. Chemical fertilization (N–P_2_O_5_–K_2_O = 88–72–36 kg ha^−1^) increased crude protein (CP) concentrations in Heuknuri and Sogang by over 30%, while reducing the soluble sugar content by 15–24%. In contrast, starch content remained relatively stable across all cultivars. Gas chromatography–mass spectrometry (GC–MS) profiling revealed that fertilization caused only modest changes in grain primary metabolism, including increased fatty acids (oleate, linoleate), alongside consistent accumulation of amino acids related to nitrogen assimilation (asparate, asparagine, glutarate, proline). Two-way ANOVA and principal component analysis (PCA) revealed that the cultivar identity, rather than fertilization, was the dominant factor shaping metabolic variation, affecting 23 of 28 detected metabolites. Notably, Betaone and Heuknuri exhibited higher overall metabolite accumulation and stable metabolic profiles across treatments, suggesting better physiological adaptation to nutrient-deficiency stress. These results indicate that NPK fertilization under reclaimed soil conditions promotes nitrogen assimilation more than carbon storage, and grain metabolic changes are largely cultivar-dependent. However, the underlying regulatory mechanisms controlling carbon–nitrogen allocation and lipid metabolism under fertilization were not fully investigated and require further multi-omics and long-term field studies.

## 1. Introduction

Barley (*Hordeum vulgare* L.) is one of the most important cereal crops globally, being widely cultivated for food, feed, and malting purposes. Its strong tolerance to adverse environments—particularly cold, drought, and salinity—allows barley to perform reliably as a winter cereal and produce stable yields in environments where other cereals such as maize or rice find it difficult to survive and prosper [1,2]. The combination of broad adaptability, relatively short growing cycles, and consistent yield potential makes barley an agronomically and economically important crop for both temperate and semi-arid agricultural systems [3].

Barley grain composition plays a key role in determining its nutritional and functional quality. Nutritionally, barley grains are composed of 54–75% starch, 8–20% protein, 11–34% dietary fiber, and 2–10% β-glucan [4,5,6]. Higher protein content is preferred in food and feed barley, while moderate protein levels are required for malting quality [6,7]. Starch serves as the major carbon and energy reserve [8], while soluble sugars and amino acids contribute to grain filling, stress responses, and malting quality [9]. Organic acids are closely associated with energy metabolism and carbon–nitrogen balance during grain development [10,11]. Therefore, changes in primary metabolite composition can reflect both the physiological status and nutritional value of barley grains under different environmental and nutrient conditions [12]. The levels of these primary metabolites, particularly carbohydrates and proteins, are strongly influenced by environmental factors, soil fertility, and crop management practices [7,13,14]. Nitrogen, phosphorus, and potassium are the most critical nutrients affecting grain filling, carbohydrate accumulation, and protein levels. N availability in soil significantly affects grain protein concentration in barley [15,16]. A higher level of N fertilizer application typically enhances yield and protein concentration in grain, but these changes are often detrimental to malting quality standards [17]. Lower availability of P and K from soil can limit the organic acid-generating pathway and, conversely, an increase in key N-related amino acids [18].

Reclaimed lands—developed from tidal flats or saline-affected areas—have been increasingly utilized for crop production in several coastal regions. These soils are typically sandy or sandy loam in texture, which promotes rapid water infiltration but also enhances the leaching of essential nutrients such as nitrogen and potassium, thereby limiting their availability during critical crop growth stages [19]. In addition, reclaimed soils often exhibit high salinity due to residual salts remaining from the reclamation process, which increases osmotic pressure, restricts water uptake, and induces osmotic stress that inhibits plant growth [20]. Moreover, reclaimed soils generally have low organic matter content, leading to poor soil structure, reduced water-holding capacity, limited microbial activity, and restricted root development, all of which further constrain crop productivity [20,21].

To overcome the above limitations, proper fertilizer management is crucial for enhancing soil fertility and ensuring stable crop production in reclaimed areas. In barley, this must be balanced against the trade-off between high yield and grain quality. Although several studies have examined the effect of nitrogen or NPK fertilization on barley growth and yield [7,13,14,15,17], little is known about how balanced NPK fertilization influences primary metabolite accumulation in barley grain grown on reclaimed soils. Understanding these relationships is essential to optimizing nutrient management and improving grain nutritional quality under such challenging soil conditions. Therefore, this study aimed to investigate the effects of NPK fertilizer on the primary metabolites of barley grains cultivated on reclaimed land, with a focus on nitrogen metabolism and central carbon metabolism.

## 2. Results

### 2.1. Effects of Fertilizer on Soluble Sugar, Starch and Total Nitrogen in Barley Grain

**Figure 1:**
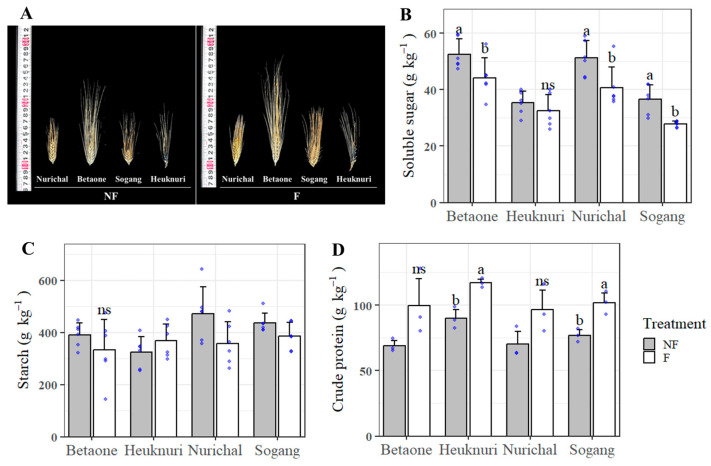
Effects of fertilizer treatment on (**A**) grain appearance, (**B**) soluble sugar, (**C**) starch, and (**D**) crude protein (CP) content in grains of four barley cultivars grown on reclaimed land. Values are means ± SD. Different letters within the same cultivar indicate significant differences between fertilizer treatments by ANOVA (*p* < 0.05) and Tukey’s test (*p* < 0.05); ns indicates not significant. Blue dots indicate the raw value.

Application of fertilizer had a significant impact on the levels of soluble sugar, starch, and crude protein (CP) in barley grains across cultivars (Figure 1, Table 1). Soluble sugar concentration was significantly higher under non-fertilized (NF) than fertilized (F) conditions in Betaone, Nurichal, and Sogang cultivars (Figure 1B), increasing by 15%, 21%, and 24%, respectively. Heuknuri showed a similar trend where NF treatment tended to have elevated soluble sugar compared to F, though the difference was not statistically significant. Moreover, the soluble sugar accumulation was higher in waxy cultivars, Betaone and Nurichal, than in the non-waxy cultivars.

Although fertilizer treatment showed a significant overall effect on starch content, *p*-value < 0.05 (Table 1), no significant differences between fertilized and non-fertilized conditions were detected within individual cultivars (Figure 1C). This suggests that starch biosynthesis in barley grain on reclaimed land is less sensitive to the fertilizer regime than soluble sugar pools. The CP content exhibited cultivar-specific responses to fertilization (Figure 1D). In Heuknuri and Sogang cultivars, fertilization (F) increased grain CP by 30–32% compared to the NF condition, indicating enhanced total nitrogen (TN) accumulation in the grain. In contrast, no significant differences in grain CP content were observed between the two treatments in the Betaone and Nurichal cultivars. However, no significant cultivar × fertilizer interaction was observed for any of the measured traits (Table 1), indicating that the fertilizer response was generally consistent across cultivars. Similar responses have been reported in barley, where grain nitrogen concentration increased under high nitrogen fertilization compared with no-nitrogen conditions [16]. This result indicates that the biosynthesis of organic compounds, such as amino acids in grain, is promoted by higher nitrogen availability. This also suggests that the difference in soluble sugar content between fertilization treatments was not due to active accumulation for osmotic regulation, but rather due to a relative lack of nitrogen for the synthesis of downstream primary metabolites such as amino acids.

### 2.2. Effects of Cultivar and Fertilizer on Metabolite Profile Change in Barley Grain

To further assess the effect of fertilizer on barley grain in reclaimed soil, GC-MS metabolite profiling was conducted, focusing on key N-related metabolism and central C metabolism. A total of 28 primary metabolites were found, consisting of 11 sugars and sugar alcohols, 10 organic acids, and seven amino acids and derivatives (Table 2). Two-way ANOVA revealed significant cultivar (C) effects (*p* < 0.05) for 23 of the 28 identified metabolites (Table 2), while fertilization (F) significantly affected only nine metabolites, including fatty acids (STA, Ole, Loa) and N-related amino acids (Pro, Pyr, Asp, Asn, Glta). Significant cultivar × fertilization (C × F) interactions were observed for only five metabolites, suggesting that most metabolic responses to fertilization were relatively consistent across cultivars.

**Figure 2:**
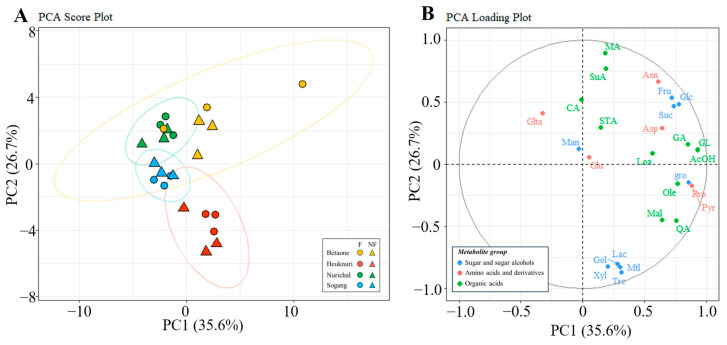
Principal component analysis (PCA) score (**A**) and loading (**B**) plots of primary metabolite profiles in barley grains under fertilized (F) and non-fertilized (NF) conditions. In the score plot, Betaone, Heuknuri, Nurichal, and Sogang are shown in yellow, red, green and blue, respectively, while circles and triangles represent fertilized (F) and non-fertilized (NF) samples, respectively. In the loading plot, sugars and sugar alcohols, amino acids and derivatives, and organic acids are shown in blue, pink, and green, respectively. PCA loading plot abbreviations: AcOH, acetate; Ara, arabinose; Asn, asparagine; Asp, aspartate; CA, citrate; Fru, fructose; GA, glycerate; GL, glycolate; Glc, glucose; Glta, glutarate; Glu, glutamate; Gol, galactinol; gro, glycerol; Lac, lactose; Loa, linoleate; MA, malate; Mal, malonate; Man, mannose; Mtl, mannitol; Ole, oleate; Pyr, pyroglutamic acid; Pro, proline; QA, quinate; STA, stearate; SuA, succinate; Suc, sucrose; Tre, trehalose; Xyl, xylitol.

Principal component analysis (PCA) explained 62.3% of the total metabolic variation, with PC1 and PC2 accounting for 35.6% and 26.7% of the variance, respectively (Figure 2). The PCA score plot showed clear separation among barley cultivars under both fertilized and non-fertilized conditions, indicating distinct metabolic responses among genotypes. The PCA loading plot indicated that glucose (Glc), fructose (Fru), sucrose (Suc), asparagine (Asn), pyruvate (Pyr), quinic acid (QA), and malate (Mal) were among the major contributors driving sample separation in PC1 and PC2, while glutamate (Glta) and mannitol (Man) contributed in the opposite direction in PC1. Notably, Heuknuri was clearly separated from the other cultivars and was associated with higher levels of carbohydrate-related metabolites, including Lac, Gol, Xyl, Mlt, and Tre. In contrast, Betaone was positioned in the positive PC1 and PC2 quadrant and was associated with nitrogen-related amino acids, particularly asparagine (Asn) and aspartate (Asp), suggesting enhanced nitrogen metabolism.

**Figure 3:**
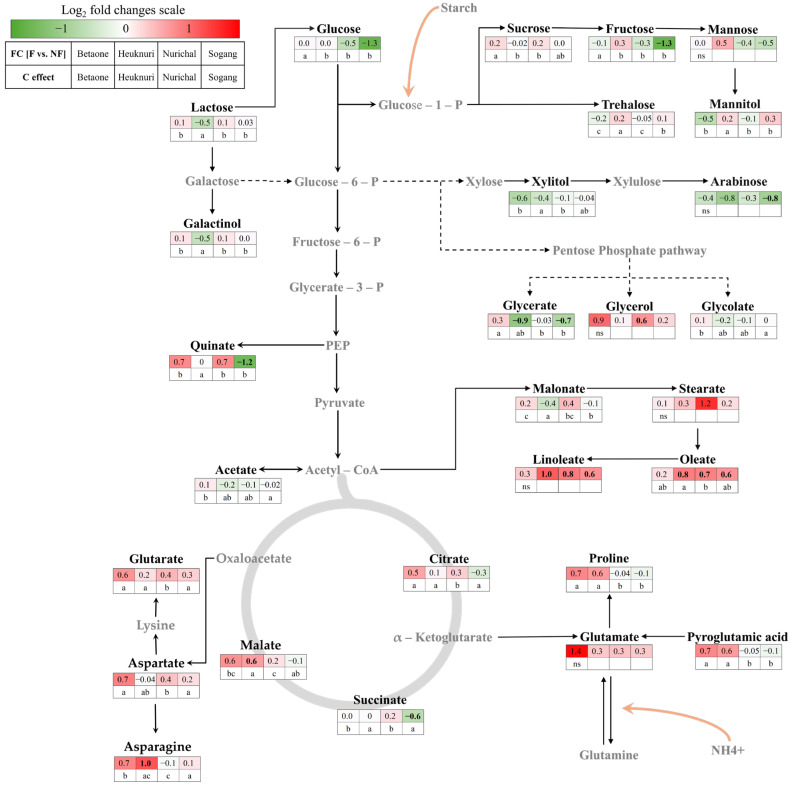
Pathway visualization of central carbon and nitrogen metabolism in barley grains affected by fertilizer application. Color intensity in the upper row represents relative changes in metabolite abundance under fertilized compared with non-fertilized conditions, with significant changes (*p* value < 0.05) in bold text. The significant letters in the lower row indicate statistical significance for the factor cultivar by ANOVA (*p* < 0.05) and Tukey’s test (*p* < 0.05). ns, not significant.

To visualize the overall metabolic responses with respect to the cultivar and fertilizer, pathway analysis and log2 fold-change (log2FC) analysis were performed (Figure 3). For each cultivar, metabolite abundances were normalized to the respective control treatment (NF) and subsequently log_2_-transformed. Therefore, positive and negative log2FC values represent relative increases and decreases in metabolite abundance following fertilization, respectively. Significant changes (*p* < 0.05) are highlighted in bold and the result of Tukey’s HSD post hoc test comparing all four cultivars is also included in Figure 3 to illustrate cultivar-specific metabolic differences. Major soluble sugars such as glucose (Glc), sucrose (Suc), and fructose (Fru) showed a stable-to-slight decrease in abundance. Similar patterns were observed for other soluble sugars, including mannose (Man) and arabinose (Ara), as well as sugar alcohols (mannitol, xylitol), indicating lower soluble sugar accumulation in grains when fertilizer was supplied. However, these reductions were more severe and statistically significant only in the Sogang cultivar. Among other carbohydrate metabolite intermediates, lactose (Lac) and galactinol (Gol) decreased only in Heuknuri, whereas the glycerol (Gro) level increased significantly in Nurichal. Moreover, Nurichal and Sogang tended toward lower overall sugar pools, while Heuknuri and Betaone retained comparatively higher levels (Figure 3). These results indicate that carbohydrate allocation and storage during grain filling are largely genotype-dependent rather than fertilizer-responsive in the reclaimed soil environment.

In contrast, organic acids from fatty acid metabolism, glycolysis, and the tricarboxylic acid (TCA) cycle generally displayed more variable responses under fertilization. Glycolysis- and PPP-related intermediates such as quinate (QA) and glycerate (GA) significantly decreased in Heuknuri and Sogang, while glycolate (GL) remained stable across cultivars. Organic acids associated with the TCA cycle largely showed unchanged trends, as seen for citrate (CA) and acetate (AcOH), except for increased malate (Mal) levels in Heuknuri and reduced succinate (SuA) levels in Sogang. In contrast to these central carbon intermediates, fatty acids derived from acetyl-CoA exhibited upward trends, with significant increases observed for linoleate (Loa) and oleate (Ole). This is somewhat in contrast to what was observed in cucumber where Loa decreased, although Ole increased upon NPK fertilization as did stearate and palmeate [22]. A study on rapeseed oil also reported that nitrogen and potassium fertilizer significantly enhance the abundance of Ole, while maintaining or slightly reducing the STA level [23], which was consistent with our result. However, Loa levels were stable even when raising the NPK fertilizer dose, contrasting with the increasing level of this FA in our study.

On the other hand, amino acid accumulation consistently increased with fertilizer application, likely due to an increase in total nitrogen content in the grain (Figure 3). Key nitrogen-related amino acids derived from the TCA cycle, such as glutamate (Glu), aspartate (Asp), and asparagine (Asn), were elevated under fertilization, particularly in Betaone and Heuknuri, although only a subset of these changes were statistically significant within cultivars. Within the γ-glutamyl pathway, pyroglutamic acid (Pyr) and Glu-derived Pro showed similar patterns with higher levels in Betaone and Heuknuri. Additionally, Nurichal had the lowest accumulation of Asp-derived amino acids, including asparagine and glutarate (Glta), in comparison to other cultivars. The changes in the major N-containing amino acids are partially similar to the situation in cucumber, where Asp and Glu were enhanced following NPK fertilization but glutamine (Gln) was not [22], whilst Gln and Asn increased upon fertilization in Purslane [24].

## 3. Discussion

The cultivar identity strongly shaped the metabolome because genetic variation determines the expression and regulation of key metabolic pathways throughout grain development. Metabolomic studies in young barley’s shoot and root consistently find cultivar-specific metabolic fingerprints even when plants are grown under the same conditions, demonstrating a strong genetic influence on metabolite profiles [25]. A study comparing wild barley (low-N-tolerant) and cultivated barley (low-N-sensitive) further showed a similar pattern, where nitrogen deficiency caused coordinated declines in TCA-related organic acids and amino acids in seedling leaves of both cultivars [26]. Evidence from other cereals also supports the dominance of genetic factors; for example, in wheat, genetic improvements were found to induce 1.4 to 7.4 times more metabolic alterations than N fertilizer supply, especially with amino acid accumulation [27]. In contrast, studies in maize have reported stronger environmental than genotypic effects on metabolomes, highlighting that genotype vs. environment effects can depend on species and context [28]. In this study, Betaone and Heuknuri exhibited higher metabolite accumulation and maintained stable profiles under both F and NF treatments (Figure 2 and Figure 3), suggesting greater tolerance to nutrient deficiencies under reclaimed soil conditions.

In contrast, fertilizer application caused small but meaningful changes in barley grain composition, mainly reflecting shifts in adjustments in carbon–nitrogen balance rather than whole metabolic reprogramming. Soluble sugars, such as Suc, Fru, and Glu, are the main carbon source for grain metabolism and are used for energy, carbon transport, and signaling during grain filling [29,30]. In our study, soluble sugars and other carbohydrate metabolites were generally reduced under fertilizer treatment (Figure 1B and Figure 2), suggesting that carbon was actively redirected toward other pathways rather than stored as free sugars. This reduction in grain sugars likely reflects the fundamental trade-off between carbon and nitrogen metabolism. Nitrogen assimilation requires substantial carbon skeletons, which can redirect available carbon from sugar pools toward amino acid synthesis. Such carbon–nitrogen competition is widely recognized because carbon metabolism provides the structural backbones required for efficient formation of nitrogen compounds. Despite these changes in soluble carbon pools, starch, the major long-term carbon storage in grains that contributes to grain weight and germination [31], remained relatively stable across treatments in our study. This is similar to previous findings under water stress [32] or drought stress [33], suggesting that abiotic stress or fertilization mainly affects short-term carbon pools rather than final carbon storage. Interestingly in Sogang, the decrease in sugar alcohols was greater than that in soluble sugars following the application of fertilizer. However, the reason why sugar alcohol levels rather than sugar levels are depleted requires further study. Intriguingly, xylitol was previously noted to be increased in cucumber upon NPK fertilization, but soluble sugars were not measured in this study [22]. By contrast, in Purslane—a plant cultivated in central Mexico—both sugars and sugar alcohols accumulated following fertilization [24].

In addition, fertilizer increased the levels of fatty acids and N-related amino acids (Figure 3). Amino acids are precursors for protein synthesis and secondary metabolites, while organic acids function in respiration, pH regulation, and carbon–nitrogen balance. The accumulation of Pro, Pyr, Asp, Asn, and Glta suggests enhanced nitrogen assimilation and amino acid metabolism under fertilizer application, reflecting shifts in carbon–nitrogen balance during grain development [34,35]. Furthermore, the significant increases in major fatty acids, including linoleate (Loa) and oleate (Ole), in three cultivars indicate that balanced nutrient supply promoted lipid biosynthesis. As reported in rapeseed, applying K increased oil concentration, effectively offsetting the reduction caused by N application [23]. Excess N promotes amino acid and protein synthesis, which competes with fatty acid biosynthesis for available carbon skeletons. In contrast, K enhances the net photosynthetic rate and increases carbon availability for fatty acid synthesis [23,36]. Additionally, K stimulates the activity of key enzymes involved in lipid biosynthesis, including acetyl-CoA carboxylase (ACCase), the rate-limiting enzyme in fatty acid synthesis [37], and phosphatidic acid phosphatase, which is essential for triacylglycerol assembly [38]. Collectively, these results suggest that combined NPK fertilization modulates carbon partitioning between nitrogen metabolism and lipid accumulation in barley grains, although the underlying regulatory mechanisms require further investigation.

Barley grain responses to fertilizer application can be strongly influenced by environmental conditions, particularly in reclaimed soils. Research from twenty-five single-year field experiments revealed that grain yield under different N rate applications significantly correlated with rainfall and negatively correlated with mean temperature, but the effect of N fertilization on grain protein concentration was not conditioned by rainfall [15]. The grain yield for the higher rainfall season was lower for the fertilized with respect to the non-fertilized treatments, while the trend was the opposite for the lower rainfall season [7]. These climate–fertilizer interactions are particularly relevant in reclaimed soils, where sandy soil textures, poor structure, and low organic matter accelerate nutrient leaching and reduce water-holding capacity. Under such conditions, rainfall events can rapidly flush nitrogen and potassium beyond the root zone [7]. Therefore, the metabolic response of barley grown on reclaimed land may be more strongly governed by cultivar tolerance to abiotic stress than by fertilizer inputs. For this reason, achieving stable grain quality and metabolic outcomes in reclaimed environments requires fertilizer strategies that account for both soil constraints and seasonal climate variability, rather than relying solely on nutrient supply.

Fertilization needs to be carefully balanced since fertilization is expensive but also the negative environmental effects of over-fertilization are dramatic and reducing these is a major sustainability goal. A further consideration is not only the yield of crops but also their quality. In this respect, a few interesting observations were made in this study. The fact that, in barley grain, sugar alcohols are reduced as opposed to the major carbohydrates probably has implications regarding quality. The ratio of carbohydrates to protein is an important quality parameter in barley, being particularly important for the malting industry as the ratio between protein and starch is highly important [7]. Moreover, nine of the proteinogenic amino acids, namely histidine, isoleucine, leucine, lysine, methionine, phenylalanine, threonine, tryptophan, and valine, cannot be synthesized de novo by humans and thus need to be acquired through our diets [39]. As such, the effects of NPK fertilizer on the barley grain content, as well as on plant yield, are also of very high interest.

However, some limitations remain, including limited insight into the regulatory mechanisms underlying the observed shifts in carbon–nitrogen balance and fatty acid metabolism, particularly the genetic and enzymatic control of carbon allocation under fertilization. Future research should focus on integrating metabolomic and transcriptomic analyses, conducting long-term field trials across diverse reclaimed soils, and evaluating a broader range of barley genotypes. Ultimately, combining appropriate cultivar selection with optimized nutrient management will be essential for improving grain quality and metabolic stability in barley grown on reclaimed land.

## 4. Materials and Methods

### 4.1. Experiment Site Information and Soil Physiochemical Properties

**Figure 4:**
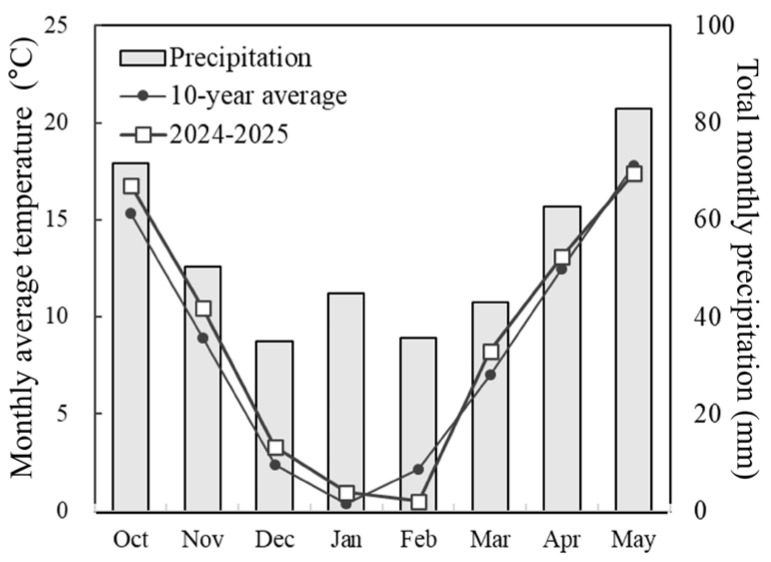
Total monthly precipitation (mm) and monthly average temperature (°C) during the barley growing seasons of 2024–2025 compared with the long-term average (2014–2023).

This study was conducted from October 2024 to May 2025 at the reclaimed experimental field of the National Institute of Crop and Food Science (NICS) in Gimje, Jeollabuk-do, Republic of Korea (35°49′44″ N, 126°41′21″ E). Weather data for the experimental period were obtained from the Korea Meteorological Administration (https://data.kma.go.kr/resources/html/en/aowdp.html; accessed on 11 September 2025) and are presented in Figure 4. The soil texture was sandy loam, and soil physicochemical properties were analyzed at three depths (0–20, 20–40, and 40–60 cm) before the experiment (Table 3). Collected soil samples were air-dried at room temperature, gently crushed, and passed through a 2 mm sieve before analysis. Soil physicochemical properties were analyzed according to standard methods of the Rural Development Administration (RDA), Republic of Korea. Soil texture was determined using the hydrometer method [40] and classified based on the USDA soil texture triangle. Soil organic matter (OM) was determined using the Walkley–Black method [41]. Available phosphate (P_2_O_5_) was analyzed using the Bray No. 1 method [42]. Exchangeable cations were extracted with 1 M ammonium acetate and quantified using an inductively coupled plasma optical emission spectrometer (ICP-OES; Avio 500, PerkinElmer, Waltham, MA, USA). Soil pH and electrical conductivity (EC) were measured using a pH meter and an EC meter after dilution with distilled water at a 1:5 (*w*/*v*) ratio. The soil EC was low, indicating minimal salinity risk. However, the soils showed low levels of organic matter (OM), available phosphate (Avail. P_2_O_5_) and exchangeable cations (K and Ca).

### 4.2. Experiment Design

Four Korean barley cultivars (*Hordeum vulgare* L.) were used as experimental materials, including 2 waxy types (Nurichal and Betaone), and 2 non-waxy types (Sogang and Heuknuri). These cultivars are classified as Korean hulled barley, which have similar requirement days for heading and grain filling. The experiment site was arranged in a split-plot design with three replications. Fertilizer treatment (NF—no fertilizer application, and F—standard fertilizer application) was assigned to the main plots, and the barley cultivar was assigned to the subplots. Each plot had an area of 30 m^2^ (3.0 m × 10.0 m). Barley seeds were sown on 19 October 2024 using the strip-seeding method with a row spacing of 40 cm and a seeding rate of 130 kg ha^−1^. Barley was harvested 220 days after sowing (DAS), and the barley grains were then collected and dried for 72 h until no change in mass occurred. Harvest maturity was determined based on spike and culm yellowing, grain hardening, and approximately 40–45 days after heading, following the recommended harvest period for grain barley in Korea.

Fertilizer was applied at the standard rates recommended by the Rural Development Administration (RDA), consisting of 88 kg N, 72 kg P_2_O_5_, and 36 kg K_2_O per hectare, using urea, superphosphate, and potassium sulfate, respectively. Nitrogen was supplied in two splits: 50% as a basal application and 50% as a topdressing at 120 DAS during the regrowth stage. Phosphorus and potassium were applied entirely as basal fertilizers.

Barley was cultivated under rainfed field conditions without supplemental irrigation during the growing seasons. Drainage channels were maintained in the field to prevent water buildup and excessive soil moisture in the plots. Before sowing, a granular soil insecticide containing ethoprophos (Mocap^®^, Dongbang Agro Corp., Seoul, Republic of Korea) was applied to prevent damage from soil insects during early crop growth. No additional plant protection chemicals were applied during the growing season because no serious pest or disease problems were observed.

### 4.3. Soluble Sugar and Starch Analysis

Soluble sugar and starch contents of barley grain were determined by the anthrone–sulfuric acid method [44]. After drying, exactly 0.1 g of powdered grain was extracted with 10 mL of 80% ethanol, and then the supernatant was collected as a fraction of soluble sugars. After evaporating, the residues (starch fraction) were dissolved in distilled water, mixed with 2 volumes of 0.2% anthrone in concentrated H_2_SO_4_, and the carbohydrate content was measured at 630 nm with a spectrophotometer (UV-1900i, SHIMADZU, Kyoto, Japan). Glucose was used as a standard. Three samples were collected for each treatment, and each measurement was conducted in duplicate.

### 4.4. Crude Protein Analysis

Crude protein content was calculated by multiplying the grain TN concentration by a nitrogen-to-protein conversion factor of 6.25. Grain TN content was determined with the Dumas combustion method using a N analyzer (Rapid MAX N exceed, Elementar Americas, Mt. Laurel, NJ, USA), following the International Organization for Standardization (ISO) method (ISO 16634-2:2016) [45]. A set of three biological replicates was collected from each treatment, and 0.3 g of each sample was used for analysis.

### 4.5. Primary Metabolites Analysis

Metabolite profiling was conducted using a modified methanol extraction and derivatization protocol [46]. In brief, 30 mg of dried barley grain was extracted with 1.0 mL of 70% (*v*/*v*) methanol and filtered through a 0.2 µm syringe filter. The filtrate (200 µL) was transferred to a 1.5 mL microtube and dried using a speed vacuum concentrator. The dried residue was dissolved in 50 µL of 20,000 ppm methyl hydroxyl chloride amine (MHCA) in pyridine and mixed with 50 µL of internal standard solution (1000 ppm fluoranthene). The mixture was incubated at 30 °C for 90 min in a thermomixer, followed by the addition of 100 µL of BSTFA (N,O-Bis(trimethylsilyl) trifluoroacetamide). After vertexing, the samples were further incubated at 60 °C for 30 min to complete derivatization. The derivatized sample was transferred to a GC glass vial with an insert and subjected to gas chromatography–mass spectrometry (GC-MS) analysis. The GC-MS system consisted of a Thermo Scientific TSQ 8000 triple quadrupole mass spectrometer coupled to a Trace 1310 gas chromatograph (Thermo Fisher Scientific, Waltham, MA, USA). Metabolite separation was achieved on a 60 m 0.25 mm id 0.25 µm DB-5 column (Agilent Technologies, Santa Clara, CA, USA) using helium as the carrier gas. Metabolites were identified by consulting the NIST Mass Spectral Library (https://chemdata.nist.gov/) based on spectral similarity and reverse similarity index (SI/RSI) values greater than 800. Relative metabolite abundances were calculated as the ratio of metabolite peak area to internal standard peak area (ISTD Area), using fluoranthene as the internal standard, and expressed as arbitrary units (a.u.) (Table S2). Three samples were collected from each treatment and duplicated GC-MS analyses of each sample were performed; thus, the reported values represent the average of the two technical measurements.

### 4.6. Statistical Analysis

Data were analyzed by ANOVA test followed by Tukey’s HSD (honest significant difference) test using the R-software version 4.5.1 (R Core Team 2016). Multiple testing correction was performed using the Bonferroni method, and adjusted *p*-values (*p*.adj) < 0.05 were considered statistically significant. Results were performed as the means ± SD. GC-MS data were normalized to the grain dry weight and the internal standard before analysis. To visualize metabolites changing between two treatments, normalization to the control and log-transformed data were performed in Microsoft Excel 5.0.

## 5. Conclusions

In this study, four Korean barley cultivars (two waxy and two non-waxy) were grown under NPK-fertilized and non-fertilized conditions in reclaimed soil to assess grain metabolic responses to nutrient management. Results show that NPK fertilizer reduced soluble sugar content by 15–24% and increased crude protein by 30–32% in responsive cultivars, indicating shifts in carbon and nitrogen metabolism. Metabolomic analysis further revealed that the fertilizer induced significant changes in several nitrogen-related amino acids and fatty acids. However, the cultivar identity had a greater influence than fertilization on grain metabolic composition, significantly affecting 23 of the 28 detected metabolites. Among the four cultivars, Betaone and Heuknuri exhibited higher metabolite accumulation and greater metabolic stability under both fertilized and non-fertilized conditions, indicating better adaptation to reclaimed soil environments. These findings improve our understanding of how nutrient management and genetic background jointly influence barley grain metabolism and provide useful information for selecting cultivars and fertilization strategies for reclaimed soils.

## Figures and Tables

**Table 1:** Results of two-way analysis of variance (ANOVA) for the effects of cultivar (C), fertilization (F), and their interaction (C × F) on soluble sugar, starch, and crude protein concentrations in barley grains cultivated in reclaimed soil. ns indicates not significant.

Trait	Cultivar (C)		Fertilizer (F)		C × F	
	F Value	*p* Value	F Value	*p* Value	F Value	*p* Value
Soluble sugar	25.971	<0.001	22.166	<0.001	1.095	0.362 (ns)
Starch	1.095	0.07 (ns)	2.545	0.047	4.21	0.097 (ns)
Crude protein	3.142	0.054 (ns)	27.42	<0.001	0.054	0.983 (ns)

**Table 2:** Effect of the combination of cultivar (C) and fertilization (F) on metabolites of barley grain in Korean reclaimed soil in the 2024–2025 season.

Metabolite		Cultivar (C)			Fertilizer (F)			C × F		
		DF	F Value	*p* Value	DF	F Value	*p* Value	DF	F Value	*p* Value
Sugar and sugar alcohols	Glucose (Glc)	3	5.05	0.012 *	1	0.04	0.849	3	0.03	0.994
	Sucrose (Suc)	3	3.61	**0.037**	1	0.27	0.612	3	0.13	0.943
	Fructose (Fru)	3	5.90	**0.007**	1	0.25	0.624	3	0.11	0.954
	Mannose (Man)	3	1.28	0.315	1	0.17	0.682	3	0.50	0.689
	Trehalose (Tre)	3	21.29	**<0.001**	1	0.36	0.559	3	0.54	0.663
	Mannitol (Mtl)	3	13.52	**<0.001**	1	0.03	0.872	3	2.18	0.13
	Lactose (Lac)	3	20.03	**<0.001**	1	0.83	0.376	3	1.73	0.201
	Galactinol (Gol)	3	20.03	**<0.001**	1	0.83	0.376	3	1.73	0.201
	Xylitol (Xyl)	3	8.01	**0.002**	1	2.75	0.117	3	0.57	0.641
	Arabinose (Ara)	3	3.15	0.054	1	24.49	**<0.001**	3	0.65	0.595
	Glycerol (Gro)	3	2.79	0.074	1	0.33	0.575	3	1.12	0.37
Organic acid	Glycerate (GA)	3	10.17	**<0.001**	1	1.72	0.208	3	2.38	0.108
	Glycolate (GL)	3	3.48	**0.041**	1	0.07	0.797	3	0.21	0.888
	Quinate (QA)	3	19.74	**<0.001**	1	0.59	0.454	3	0.53	0.666
	Malonate (Mal)	3	33.63	**<0.001**	1	0.10	0.754	3	4.76	**0.015**
	Stearate (STA)	3	2.71	0.08	1	8.71	**0.009**	3	3.39	**0.044**
	Oleate (Ole)	3	9.77	**<0.001**	1	22.97	**<0.001**	3	1.66	0.216
	Linoleate (Loa)	3	1.06	0.393	1	27.90	**<0.001**	3	1.24	0.328
	Acetate (AcOH)	3	3.52	**0.039**	1	0.09	0.764	3	0.21	0.889
	Citrate (CA)	3	9.48	**<0.001**	1	0.86	0.367	3	0.76	0.531
	Succinate (SuA)	3	16.10	**<0.001**	1	0.28	0.606	3	2.03	0.151
	Malate (MA)	3	15.19	**<0.001**	1	4.46	0.051	3	1.57	0.235
Amino acids and derivatives	Glutamate (Glu)	3	1.92	0.167	1	3.20	0.093	3	1.22	0.335
	Proline (Pro)	3	29.69	**<0.001**	1	8.81	**0.009**	3	3.64	**0.036**
	Pyroglutamic acid (Pyr)	3	29.69	**<0.001**	1	8.81	**0.009**	3	3.64	**0.036**
	Aspartate (Asp)	3	8.33	**0.001**	1	5.54	**0.032**	3	2.19	0.129
	Asparagine (Asn)	3	49.71	**<0.001**	1	11.67	**0.004**	3	5.00	**0.012**
	Glutarate (Glta)	3	12.64	**<0.001**	1	7.76	**0.013**	3	0.65	0.595

* *p*-values were obtained from two-way ANOVA, and significant effects (*p* < 0.05) are shown in bold.

**Table 3:** Soil physicochemical properties before the experiment at different depths.

Soil Depth (cm)		0–20	20–40	40–60	Optimum Range *
Exchangeable cations (cmol_c_ kg^−1^)	K	0.34 ± 0.10	0.38 ± 0.11	0.39 ± 0.01	0.50–0.80
	Ca	3.3 ± 0.1	2.7 ± 0.0	2.2 ± 0.0	5.0–6.0
	Mg	1.3 ± 0.1	1.5 ± 0.1	1.6 ± 0.1	1.5–2.0
	Na	0.06 ± 0.02	0.07 ± 0.02	0.09 ± 0.01	-
Avail. P_2_O_5_ (mg kg^−1^)		34.3 ± 0.4	19.9 ± 1.0	16.5 ± 0.9	150–250
OM (g kg^−1^)		8.0 ± 0.4	4.8 ± 0.1	4.1 ± 0.1	20–30
pH (1:5)		6.8 ± 0.1	7.4 ± 0.1	7.4 ± 0.1	6.0–7.0
EC (dS m^−1^)		0.36 ± 0.01	0.35 ± 0.01	0.33 ± 0.01	-

* Optimum range indicates the soil criteria for the upland recommended by the Rural Development Administration (RDA), Republic of Korea [43].

## Data Availability

The original contributions presented in this study are included in the article. Further inquiries can be directed to the corresponding authors.

## Supplementary Materials

The following supporting information can be downloaded at: https://www.mdpi.com/article/10.3390/plants15121780/s1, Table S1. Effect of the fertilization on metabolite changes of barley grain, Table S2: Relative metabolite abundances (peak area/internal standard area) determined by GC–MS in barley grains under fertilized (F) and non-fertilized (NF) conditions.

## Abbreviations

The following abbreviations are used in this manuscript:

N	Nitrogen
P	Phosphorus
K	Potassium
F	Fertilized
NF	Non-fertilized
GC-MS	Gas chromatography–mass spectrometry
TCA	Tricarboxylic Acid cycle
